# Numerosity estimation in children with ADHD across space, time, vision and action

**DOI:** 10.3389/fnhum.2026.1940478

**Published:** 2026-09-04

**Authors:** Giovanni Anobile, Sofia Ditano, Silvia Martelli, Federica Ricci, Eleonora Bieber, Chiara Pfanner, Francesca Tinelli

**Affiliations:** 1 Department of Neuroscience, Psychology, Pharmacology and Child Health, University of Florence, Florence, Italy; 2 Department of Developmental Psychiatry and Psychopharmacology, IRCCS Fondazione Stella Maris, Pisa, Italy; 3 Department of Developmental Neuroscience, IRCCS Fondazione Stella Maris, Pisa, Italy

**Keywords:** ADHD, approximate number system, children, mathematical abilities, numerical cognition, numerosity, sensorimotor

## Abstract

Children with ADHD often experience difficulties in mathematics, but the mechanisms underlying these difficulties remain unclear. One possibility is that they reflect a basic impairment in the “number sense,” that is, the ability to approximate the number of objects and temporal events without counting. Here, we tested this hypothesis by asking children with ADHD (*N* = 20) and typically developing controls (*N* = 43) to verbally estimate briefly presented dot arrays, temporal sequences of flashes, and self–generated hand actions (button presses). Performance was quantified in terms of precision (Weber fraction) and accuracy (absolute bias). In the unadjusted analysis, children with ADHD showed lower overall estimation precision than controls. The group difference disappeared after adjustment for age, reasoning abilities, and mathematical performance. No group differences emerged for estimation accuracy. Taken together, these findings provide no clear evidence of impaired approximate numerosity processing in ADHD and are consistent with the possibility that mathematical difficulties do not primarily arise from a core deficit in the non–symbolic number sense.

## 1 Introduction

Numerosity perception, the ability to estimate the number of objects/events without counting, is considered a core numerical competence (Feigenson et al., 2004). This ability allows humans to rapidly extract approximate quantitative information from the environment and has been proposed as a domain–specific foundation for the development of mathematical skills (Piazza, 2010). Numerosity processing is typically assessed using either comparison tasks, which require judging the more numerous of two arrays (which more numerous?), or estimation tasks, which require mapping approximate quantities onto verbal number labels (how many?). Performance is typically indexed by Weber fraction, a measure of precision, and individual differences in precision correlate with mathematical competence (Chen and Li, 2014; Halberda et al., 2008; Schneider et al., 2017), supporting the hypothesis that non–symbolic number sense may scaffold symbolic mathematics (Piazza, 2010). In developmental dyscalculia, impairments in visual spatial numerosity processing, have frequently been observed and interpreted as evidence that an early weakened number sense could contribute to later mathematical difficulties (Anobile et al., 2018b; Decarli et al., 2023; Mazzocco et al., 2011; Piazza et al., 2010). Dyscalculia is not the only disorder associated with low math skills. ADHD has been often associated with reduced academic achievement, including lower mathematical performance (Ganor-Stern and Steinhorn, 2018; Kanevski et al., 2022; Kaufmann and Nuerk, 2008; Orbach et al., 2020; Tosto et al., 2015; von Wirth et al., 2021; Zentall et al., 1994). While the mechanisms underlying these difficulties remain unclear, one possibility is that they reflect a deficit in basic numerosity representations, analogously to what has been proposed for dyscalculia. Previous work provided a first attempt to test this idea (Anobile et al., 2022). Using a non–symbolic comparison task with visual–spatial numerosities (dots), the authors found no evidence of impaired numerosity precision and accuracy in children with ADHD, despite lower mathematical performance. This evidence suggested that mathematical difficulties in ADHD might not originate from an impaired visual number sense. However, in that experiment participants were required to compare spatial numerosities, not to explicitly map numerosity onto verbal labels. Numerosity estimation and comparison do not necessarily provide equivalent measures of numerosity perception. Krueger (1984) and Guillaume et al. (2016) both showed that numerosity comparison performance is weakly related, or even unrelated, to performance measured with verbal estimation tasks suggesting that the two procedures rely on (at least partly) independent processes. Accordingly, preserved performance in the numerosity comparison task mentioned above (Anobile et al., 2022) leaves open whether ADHD–related difficulties may emerge in verbal estimation tasks, when approximate quantities must be explicitly mapped onto numerical labels.

Moreover, that previous work was restricted to visual spatial numerosity, but a growing body of evidence suggests that the number sense should be considered a generalized mechanism working over both spatial and temporal numerosity formats. Correlational studies on children showed links between numerosity precision measured with visual spatial (dots) and temporal (flashes) numerosity, suggesting shared resources (Anobile et al., 2018a; Anobile et al., 2026). Similar results were found with psychophysical adaptation paradigms in adults (Arrighi et al., 2014; Burr et al., 2018). Converging neurophysiological and neuroimaging findings indicate that spatial and temporal numerical formats recruit partially overlapping representations, with both format–dependent and format–independent responses identified in the human intraparietal sulcus, the monkey parietal cortex, and the crow endbrain (Ditz and Nieder, 2020; Dormal et al., 2010; Nieder et al., 2006). Nevertheless, other neuroimaging evidence indicates that the posterior parietal cortex may contribute specifically to visual–spatial numerosity, whereas visual–temporal numerosity may rely more strongly on temporal regions (Cavdaroglu and Knops, 2019). Together, these findings suggest that spatial and temporal numerical formats are processed through partly shared mechanisms.

A more recent line of research extended this idea to the motor system. It has been suggested that the number sense is able to integrate the numerosity of sensory inputs (objects and temporal events) with the number of self–generated actions (e.g., up–down hand tapping) (Anobile et al., 2021a). Here, “self–generated actions” refers to movements actively performed by the individual; the relevant dimension is the number of action repetitions, not their form or meaning. In line with this, recent evidence in children shows positive correlations in estimation precision across these three numerical formats (Anobile et al., 2026), and studies with adults provide converging evidence for the existence of a sensorimotor numerosity system (Anobile et al., 2016, 2024; Benedetto et al., 2024; Petrizzo et al., 2025; Yang et al., 2024). These behavioural findings are complemented by neurophysiological evidence. Sawamura et al. (2002) first identified neurons in the monkey parietal cortex that were selective to the ordinal position of repeated hand movements, thereby tracking the number of actions. Transient inactivation of those neurons in the monkey cortex (by muscimol injection) induced numerical errors, suggesting a causal role (Sawamura et al., 2010). Kirschhock and Nieder (2022) subsequently identified neurons in the crow telencephalon tuned to the impending number of pecks required to match a visually presented numerosity (dot arrays). More recently, Seidler et al. (2026) showed that neurons in the macaque ventral intraparietal cortex encode the intended number of hand movements during motor planning. Imaging studies in humans also suggest parietal mechanisms tuned to the number of observed actions (Sawamura et al., 2020). Taken together, these psychophysical, neurophysiological and imaging findings provide converging evidence for motor and sensorimotor mechanisms linking the numerosity of external stimuli to the planning, observation and production of actions consistent with sensorimotor accounts of numerical cognition (Anobile et al., 2021a; Prabhu et al., 2026; Ranzini et al., 2022, 2025; Sixtus et al., 2023).

The literature on the role of different numerosity formats on mathematical learning is however still scarce. While the link between visual spatial numerosity and math skills seems relatively consolidated (Chen and Li, 2014; Schneider et al., 2017), one previous work found no links between estimation of visual flashes and math abilities in children (Anobile et al., 2018a). One more recent study replicated that null correlation and showed that children with higher precision in estimating the number of self–generated hand actions (without counting) were also those with higher mental calculation abilities (Anobile et al., 2026) suggesting that motor but not visual temporal numerosity is linked with math abilities in children.

Building on this evidence, the current study tested whether the previously observed absence of an ADHD–control difference in a visual–spatial numerosity comparison task extends to verbal estimation tasks across three conditions: simultaneously presented visual–spatial dot arrays, sequential visual flashes, and sequential motor actions. The main aim was therefore to assess the cross–task and cross–format generality of the previously observed results (Anobile et al., 2022).

To this aim, we asked children with and without ADHD to verbally estimate visual–spatial arrays of dots, temporal sequences of flashes, and self–generated sequences of button presses. Mathematical ability, verbal and non–verbal reasoning skills were also measured by age standardized tests. If mathematical difficulties in ADHD are linked to a deficit in the number sense, group differences should emerge in one or more of these numerosity tasks. By contrast, a preserved number sense would suggest that mathematical difficulties associated with ADHD could be linked to other cognitive factors.

## 2 Methods

### 2.1 General procedure

All participants completed three numerosity estimation tasks, mental calculation tasks, and two reasoning tasks (one verbal and one non–verbal). The temporal order of tasks was pseudorandomized, except that the visual–temporal estimation task always followed the motor estimation task, although not necessarily immediately. Participants were tested individually in a single session, lasting approximately 1.5 h (with breaks). Visual stimuli were created with Psychophysics toolbox for Matlab (Brainard, 1997; Kleiner et al., 2007; Pelli, 1997) and displayed on a 60 Hz—15″ screen monitor placed at a viewing distance of approximately 57 cm. Before testing, parents provided informed consent. Procedures were approved by the local ethics committee (Florence Meyer’s Hospital, n. 248/2020 ID- DNATN).

### 2.2 Participants

The control dataset was recently reported in Anobile et al. (2026). For the current study, we re–analysed the original data from this sample and compared it to a newly collected clinical group (with diagnosis of ADHD). Sixty–eight participants were initially tested. Four control participants and one ADHD participant were then excluded for failing to follow instructions in one or more of the numerosity tasks and/or for not completing one or more of the neuropsychological tasks. Statistical analyses were thus performed on sixty–three participants: 20 with a diagnosis of ADHD (7 female, average 11 years. 10 months, SD 2 years. 7 months, min 7 years. 7 months, max 17 years. 6 months) and 43 with neurotypical development (27 female, average 11 years. 9 months, SD 2 years. 4 months, min 8 years. 9 months, max 17 years. 9 months). The ADHD and control groups did not differ in age (*t*_(61)_ = 0.1, *p* = 0.92, LBF = −0.56). Clinical diagnosis of ADHD was performed by a specialized clinical team in the Stella Maris Hospital (Pisa, Italy) based on the DSM–5. ADHD symptoms were assessed by the ADHD Rating Scale–5, completed by parents (DuPaul et al., 2019; see Table 1). Among the 20 participants with ADHD, 14 met criteria for the combined presentation and 6 for the predominantly inattentive presentation. Participants with ADHD had no neurological or sensory deficits and no current or past pharmacological treatment. Four participants with ADHD also met DSM–5 criteria for a learning disorder: one dyslexia, two dyscalculia, and one both dyslexia and dyscalculia. These participants were retained in the primary analyses. A control analysis was additionally conducted after excluding the three participants with dyscalculia.

**Table 1:** Descriptive statistics.

	Group	Mean	St Dev
Age	ADHD (*N* = 20)	11 years. 10 months	2 years. 7 months
	Controls (*N* = 43)	11 years. 9 months	2 years. 4 months
Similarities	ADHD (*N* = 20)	11.35	2.306
	Controls (*N* = 41)	13.805	3.106
Matrices	ADHD (*N* = 20)	10.4	2.787
	Controls (*N* = 41)	13.317	2.223
Full IQ scale	ADHD (*N* = 20)	98.7	13.2
	Controls	Not available	
ADHD Rating Scale–5 (percentiles)	ADHD, Attention (*N* = 20)	89	13
	ADHD, Hyperactivity/impulsivity (*N* = 20)	80	18
	ADHD, Total score (*N* = 20)	82	19
	Controls	Not available	
Math index (Z–score)	ADHD (*N* = 20)	−0.501	1.054
	Controls (*N* = 43)	0.392	0.736
WF Dots	ADHD (*N* = 20)	0.176	0.052
	Controls (*N* = 43)	0.148	0.049
WF Flashes	ADHD (*N* = 20)	0.182	0.08
	Controls (*N* = 43)	0.149	0.07
WF Actions	ADHD (*N* = 20)	0.20	0.086
	Controls (*N* = 43)	0.18	0.077
Abs Error Dots	ADHD (*N* = 20)	1.236	0.657
	Controls (*N* = 43)	1.454	1.034
Abs Error Flashes	ADHD (*N* = 20)	1.418	0.596
	Controls (*N* = 43)	1.275	0.616
Abs Error Actions	ADHD (*N* = 20)	1.691	0.929
	Controls (*N* = 43)	1.391	0.956

The control group consisted of a sample of neurotypical children matched for chronological age with no medical and neuro–psychiatric history and no learning difficulties (reported by parents).

### 2.3 Psychophysical tasks

The experimental tasks and stimuli for the control group were identical to those described in Anobile et al. (2026). The clinical sample with ADHD was tested using the same tasks, apparatus, and procedure. On each task, participants received 6 practice trials (one for each target number: N 10, 11, 12, 13, 14 and 15) with trial–by–trial feedback (a digit representing the physical number of produced actions or the number of presented dots or flashes; training data were excluded from analyses) and 48 test trials with no feedback (8 trials per target number, randomly selected trial–by–trial). Participants verbally estimated the number of actions/dots/flashes, and responses were entered by an experimenter via keyboard.

#### 2.3.1 Motor numerosity

Participants fixated a central red circle (diameter 10°) until it turned green: the go–signal. They then tapped the spacebar with their dominant hand at a comfortable rate. The software monitored the number of presses in real time and determined the stop signal: the disappearance of the green circle and a beep sound. Afterward, participants reported the estimated number of self–generated actions. Counting was prevented by articulatory suppression (repeating the syllable “ba” as quickly as possible).

#### 2.3.2 Visual–temporal numerosity

Participants fixated a central black spot, then a central stream of white flashes (100% contrast, 10° diameter) was presented. At the end of the sequence, participants reported the perceived number of flashes. Stimuli were built from the sequences produced in the motor condition. In that task each keypress and inter–press interval was recorded as a distinct numerical value, for each participant and trial. These temporal traces were subsequently converted into visual stimuli, with the keypress replaced by white disks. Sequences were presented in the same temporal order as originally produced in the motor task. Counting was prevented by articulatory suppression (repeating the syllable “ba” as quickly as possible). Participants were not informed that the visual sequences reproduced the temporal structure of the sequences they had previously generated in the motor task.

#### 2.3.3 Visual–spatial numerosity

In each trial, participants fixated a central black point, then a central array of non–overlapping black dots was briefly presented (500 ms). The dots (0.5° diameter) appeared in random positions within a virtual circle (14° diameter). Participants estimated the perceived number of dots.

### 2.4 Mathematical abilities

Participants with ADHD completed a full age–appropriate battery for the diagnosis of dyscalculia: BDE2 (Biancardi et al., 2016, 14 participants), ACMT3 (Cornoldi et al., 2020, 2 participants), or MT3 (Baldi et al., 2017, 4 participants). Control participants completed two age– and grade–appropriate mathematics subtests from either BDE2 (39 participants) or MT3 (4 participants), as detailed below. The two youngest participants with ADHD completed the ACMT3 Mental Calculation and Arithmetic Facts subtests because the other batteries were not suitable for their age. These subtests were selected to assess mathematical abilities as closely comparable as possible to those measured in the older participants. For each subtest, the number of correct responses obtained within the time limit was converted into an age-standardized z–score using the normative data provided in the corresponding test manual. For each participant, the composite Math Index was calculated by averaging the two age-standardized z–scores obtained from the relevant age–appropriate subtests. Subtests administered only as part of the full ADHD diagnostic battery were not included. Although the specific subtests varied according to age and test battery, all component scores were expressed on the same normative z–score metric before averaging. The Math Index was lower in the ADHD group than in the control group (*t*_(61)_ = 3.841, *p* < 0.001, LBF = 1.93, see Table 1 for descriptive statistics).

*Rapid calculation (BDE2, grades 4–9, N41)*: Participants solved as many operations as possible within 2 min. Operations varied in formats and difficulty (additions, subtractions, multiplications, and divisions), were presented on paper and had to be solved mentally. *Mental calculation (BDE2, grades 3–9, N53)*: The experimenter read (one−by−one) 9 additions and 9 subtractions (one to three digits). Verbal response time limit was 30″. *Mental multiplication (BDE2, 3rd grade only, N12):* The experimenter read (one−by−one) 18 single−digit multiplications. Verbal response time limit was 3″. *Mental calculation (MT−3, from 9th grade, N8)*: The experimenter read 8 operations (additions, subtractions, multiplications, and divisions). Verbal response time limit was 60″. *Arithmetic facts (MT−3, from 9th grade, N2)*: The experimenter read (one–by–one) 27 operations (e.g., 5 × 7, 100 ÷ 2, 2 + 7, √49). Verbal response time limit was 3″. *Mental calculation (ACMT3, 2nd grade only, N2)*: The experimenter read (one-by-one) 8 operations (additions and subtractions). Verbal response time limit was 30″. *Arithmetic facts (ACMT3, 2nd grade only, N2)*: The experimenter read 8 simple one−digit operations. Verbal response time limit was 3″.

### 2.5 Reasoning abilities

Participants with ADHD completed a full age–appropriate Wechsler scale. Controls reasoning abilities were assessed by two sub − scales of the same batteries: similarities and matrix reasoning. In the similarities task, participants had to describe what two words had in common (e.g., milk and water), an index of verbal intelligence. In the matrix reasoning task, participants had to complete visual patterns by selecting the missing element from five alternatives, an index of non − verbal reasoning. Raw scores were converted into age standardized scores, according to the test manual. Verbal and non–verbal reasoning abilities were both lower in the ADHD group compared to controls (*t*_(59)_ = 3.1, *p* = 0.003, LBF = 1.1 and *t*_(59)_ = 4.368, *p* < 0.001, LBF = 2.59 respectively, see Table 1 for descriptive statistics).

## 3 Data analysis

### 3.1 Numerosity estimation precision and accuracy

For each participant, task, and target numerosity, we computed mean response, Weber fraction (Wf), and absolute bias (AB). Wf indexed precision and was calculated as the response standard deviation divided by target numerosity. Wfs were then averaged across tested numerosities, yielding one mean Wf for each participant and task. AB indexed accuracy and was calculated, for each target numerosity, as the absolute value of the mean signed difference between estimated and physical numerosity. As for Wfs, values were then averaged across target numerosities for each participant and task. Across tasks and groups, mean Wf and AB were non–normally distributed (Shapiro–Wilk *p* < 0.05), but normality was achieved after log10 transformation (all *p* > 0.05). Analyses were therefore conducted on log10-transformed values.

Group differences were tested by linear mixed–effects models, with Wf and AB as dependent variables. Group (ADHD, control) was entered as a between–subject factor, Task (dots, flashes, actions) as a within–subject factor, and participants as random intercepts. Age, Similarities, Matrices, and Math Index were included (or not) as predictors. Given the small ADHD sample, only the Task × Group interaction was retained, as it addressed the primary research question.

Frequentist and approximate Bayes factors were computed from post–hoc *t* statistics (provided by the mixed models) following Faulkenberry (2018). Bayes factors were reported as log10(BF), with LBF > 0.5 indicating substantial evidence for a group difference and LBF < −0.5 substantial evidence against it (Jeffreys, 1961). Model–adjusted standardized group contrasts (d_adj_) and their 95% confidence intervals were calculated on the log10 scale by dividing the model–estimated ADHD–control differences and their unadjusted confidence limits by the residual standard deviation of the corresponding mixed–effects model. Bonferroni correction was applied to the *p* values of the three task–specific group comparisons by multiplying each unadjusted *p* value by three (ADHD vs. control for dots, flashes and actions), with adjusted values capped at 1 (p_bonf_). Confidence intervals were not adjusted for multiple comparisons. Models were run in Jamovi 2.6.45.0; Bayes factors and psychophysical parameters were computed in MATLAB R2023b.

### 3.2 Analysis of motor sequences

This analysis had two aims: to examine whether participants with ADHD and controls differed in the temporal characteristics of their motor productions and, because each visual flash sequence reproduced the temporal structure of the corresponding participant’s motor sequence, to determine whether motor differences between groups resulted in different temporal structures of the visual stimuli. Motor responses were recorded frame by frame at 60 Hz and coded as either unpressed or pressed. A tap was identified as a transition from the unpressed to the pressed state; consecutive pressed frames were therefore treated as a single sustained tap. Three participant–level parameters were calculated. For each trial, sequence duration was defined as the time from the first pressed frame to the last pressed frame, with both frames included. Tapping rate was then calculated by dividing the number of detected taps in that trial by its sequence duration and was expressed in Hz. Mean tapping rate and mean sequence duration were obtained for each participant by averaging the corresponding values across all trials. Inter–event intervals (IEIs) were calculated as the elapsed time, in milliseconds, between the onset of one tap and the onset of the next tap within the same trial. All IEIs from all trials were pooled separately for each participant. The IEI coefficient of variation was calculated by dividing the standard deviation of these pooled IEIs by their mean and provided a dimensionless measure of temporal variability, with higher values indicating a less regular tapping pattern. The three parameters—mean tapping rate, mean sequence duration, and IEI coefficient of variation—were compared between the ADHD and control groups using independent samples *t*–tests. Motor parameters were computed in MATLAB R2023b.

## 4 Results

**Figure 1:**
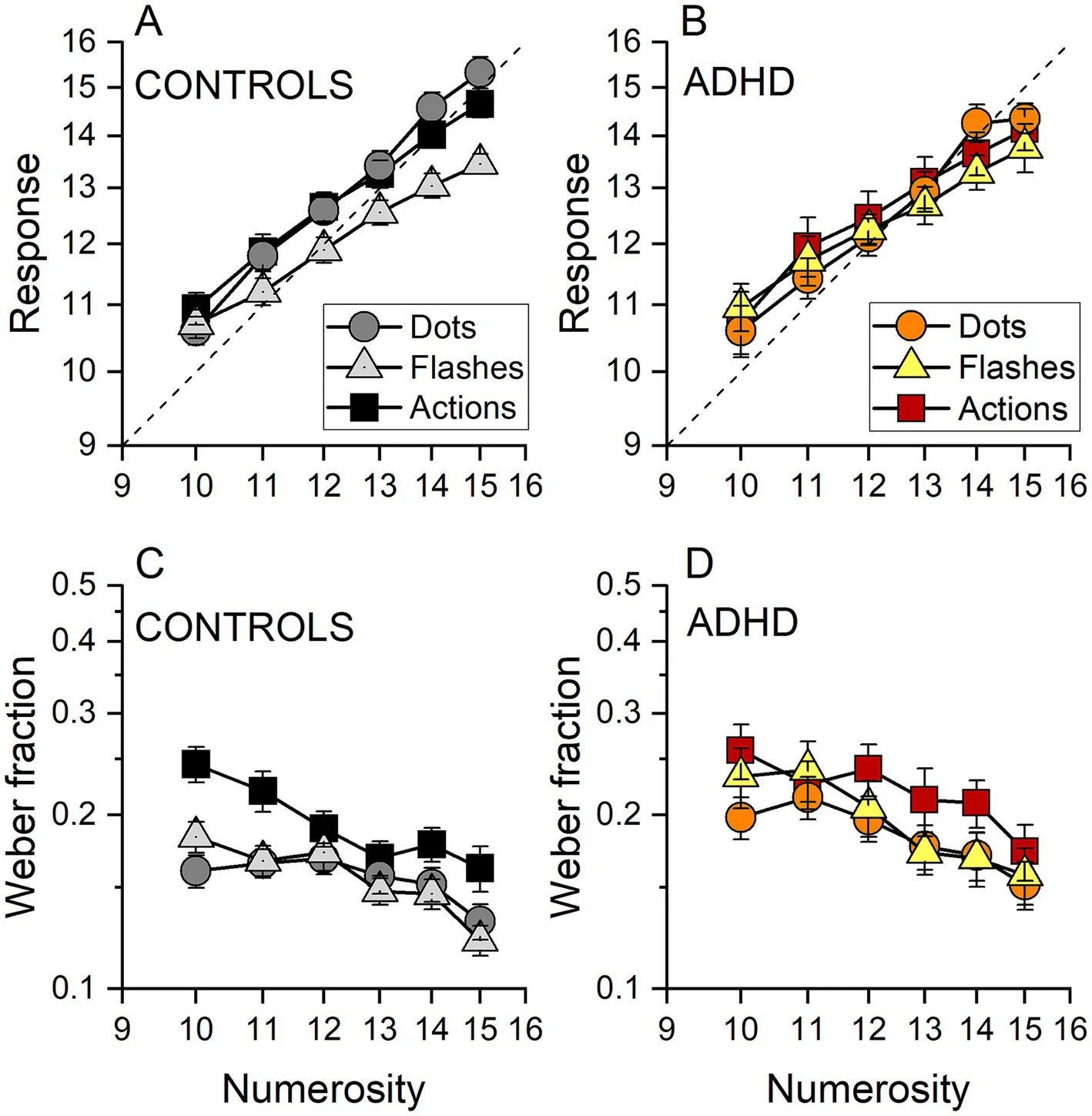
Mean responses and Weber fractions across numerosities in the control and ADHD groups. **(A,B)** Show estimates as a function of target numerosity for controls **(A)** and ADHD **(B)** in the three tasks (dots, flashes, actions). **(C,D)** Show Weber fractions across numerosity levels for controls **(C)** and ADHD **(D)**.

All participants were able to perform the psychophysical numerosity tasks, with responses scaling linearly with target numerosity (Figures 1A,B) and Wfs roughly constant across numerosity levels (Figures 1C,D). On visual inspection there are no obvious between groups differences in both accuracy (responses) and precision. We then quantified between–group differences by means of linear mixed models, predicting Wfs or AB.

### 4.1 Numerosity estimation precision

**Figure 2:**
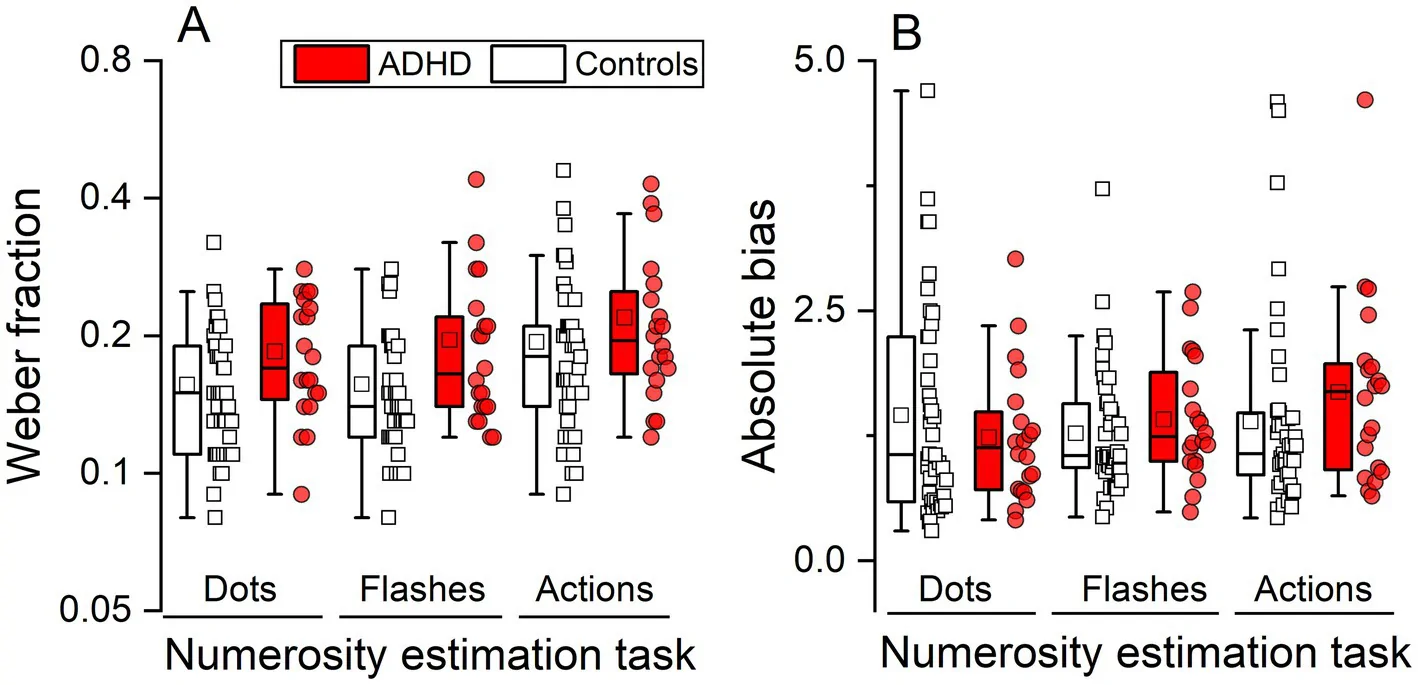
Participant–level numerosity estimation performance across tasks and groups. **(A)** Shows Weber fractions (precision), and **(B)** shows absolute biases (accuracy) for the dots, flashes, and actions estimation tasks in the control and ADHD groups. Each point represents one participant’s mean value across the tested numerosities. Boxes span the 25th to 75th percentiles, horizontal lines indicate the median, and whiskers indicate values within 1.5 times the interquartile range.

We first evaluated a model predicting Wfs without including covariates (see Figure 2A for individual subject data). Wfs varied significantly across the three tasks [*F*(2,122) = 10.83, *p* < 0.001], with the motor task yielding the lowest precision. A difference between groups was observed. Children with ADHD differed from controls in their overall level of estimation precision [*F*(1,61) = 5.11, *p* = 0.027]. Across tasks the mean Wf in the ADHD group was 0.188, 95% CI = [0.16, 0.21], while in the control group was 0.16, 95%CI = [0.14, 0.17]. The corresponding LBF was 0.2 suggesting only anecdotal evidence for a group difference. The interaction between Task and Group was not significant [*F*(2,122) = 0.371, *p* = 0.69], suggesting that the difference was similar across tasks. We then tested differences for each task by post–hoc *t*–tests. The contrast do not survive Bonferroni correction and Bayesian analyses favoured the null hypothesis of no group difference over the alternative (ADHD vs. Controls; Dots: *t*_(112)_ = 1.987, p_bonf_ = 0.147, d_adj_ = 0.82, 95% CI = [0, 1.63], LBF = −0.42; Flashes: *t*_(112)_ = 2.261, p_bonf_ = 0.078, d_adj_ = 0.93, 95% CI = [0.12, 1.74], LBF = −0.29; Actions: *t*_(112)_ = 1.47, p_bonf_ = 0.432, d_adj_ = 0.6, 95% CI = [−0.21, 1.42], LBF = −0.64).

**Figure 3:**
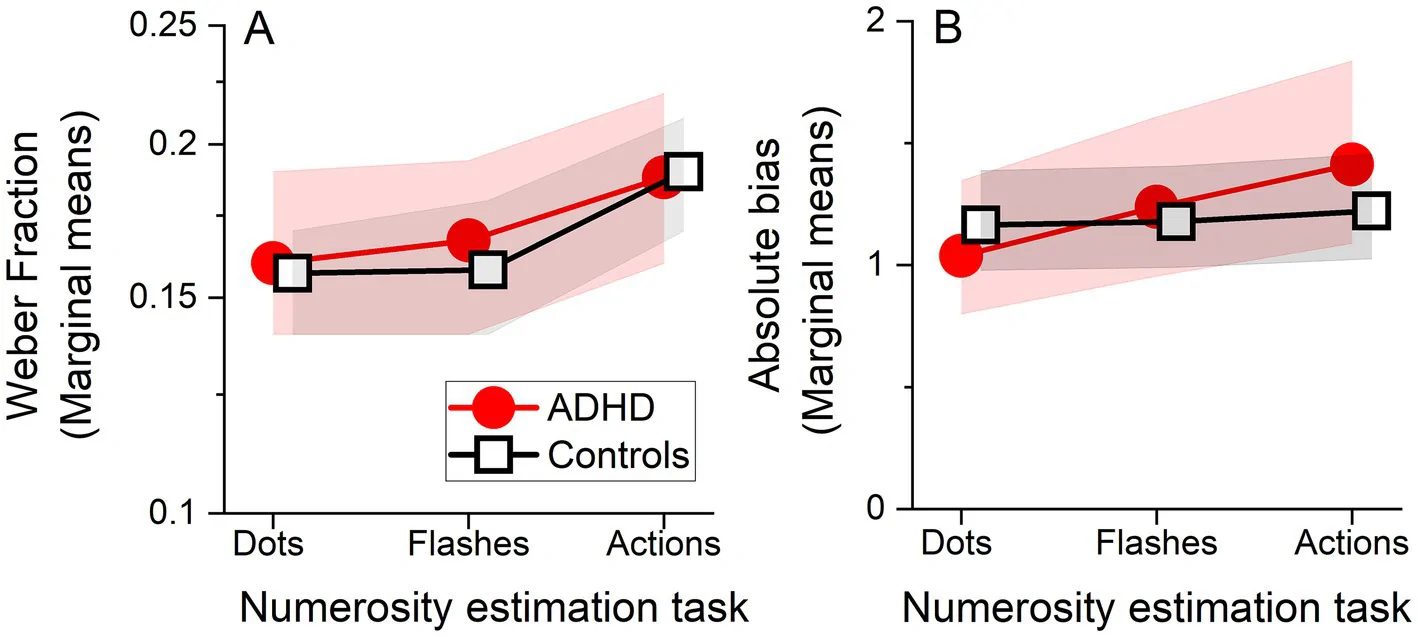
Estimated marginal means from the linear mixed models across tasks in the ADHD and control groups. **(A)** Shows Weber fraction (precision), and **(B)** shows absolute bias (accuracy) for dots, flashes, and action estimation tasks. Shaded areas represent 95% CI.

The same model was then evaluated including all the covariates (Table 2; Figure 3A). The analysis confirmed an effect of task. Wfs varied significantly across the three tasks [*F*(2,118) = 10.34, *p* < 0.001], with the motor task yielding the lowest precision (Table 3; Figure 2A). No overall difference between groups was observed. Children with ADHD did not differ from controls in their overall level of estimation precision [*F*(1,55) = 0.05, *p* = 0.818]. The interaction between Task and Group was also not significant [*F*(2,118) = 0.348, *p* = 0.707], suggesting that both groups showed a similar pattern of estimation precision across tasks, confirmed by post–hoc *t*–test (ADHD vs. Controls; Dots: *t*_(92.5)_ = 0.27, p_bonf_ = 1, d_adj_ = 0.12, 95% CI = [−1.15, 1.36], LBF = −0.88; Flashes: *t*_(92.5)_ = 0.51, p_bonf_ = 1, d_adj_ = 0.23, 95% CI = [−1.05, 1.46], LBF = −0.86; Actions: *t*_(92.5)_ = −0.19, p_bonf_ = 1, d_adj_ = −0.08, 95% CI = [−1.36, 1.15], LBF = −0.89).

**Table 2:** Linear mixed-effects model.

Dependent variable: precision (Weber fraction)
	*F*	df	df (res)	*p*
Task	10.34	2	118	< 0.001
				
Group	0.05	1	55	0.818
Similarities	0.376	1	55	0.542
Matrices	1.65	1	55	0.204
Age	5.35	1	55	0.024
Math index	10.295	1	55	0.002
Task * group	0.348	2	118	0.707

Dependent variable: precision (Absolute bias)
	*F*	df	df (rest)	*p*
Task	1.75	2	118	0.178
Group	0.044	1	55	0.834
Similarities	0.002	1	55	0.96
Matrices	0.368	1	55	0.54
Age	0.01	1	55	0.9
Math index	4.98	1	55	0.03
Task * group	0.95	2	118	0.389

Covariates: age, similarities, matrices, math index.

**Table 3:** Estimated marginal means, standard errors (SEs), degrees of freedom (df), and 95% confidence intervals (CIs) from the linear mixed-effects models for numerosity estimation precision (Weber fractions) and accuracy (Absolute bias), by task and group.

Estimated marginal means – precision (Weber fractions)						
Task	Group	Mean	SE	df	95% CI	
					Lower	Upper
Dots	ADHD	0.161	0.012	97.3	0.138	0.187
Dots	Controls	0.157	0.008	103.7	0.142	0.173
Flashes	ADHD	0.167	0.013	97.3	0.143	0.194
Flashes	Controls	0.158	0.008	103.7	0.143	0.175
Actions	ADHD	0.188	0.014	97.3	0.161	0.219
Actions	Controls	0.191	0.010	103.7	0.173	0.212

Estimated marginal means – accuracy (Absolute bias)						
Task	Group	Mean	SE	df	95% CI	
					Lower	Upper
Dots	ADHD	1.038	0.136	144	0.800	1.346
Dots	Controls	1.164	0.102	152	0.979	1.387
Flashes	ADHD	1.239	0.163	144	0.955	1.607
Flashes	Controls	1.178	0.103	152	0.991	1.406
Actions	ADHD	1.413	0.185	144	1.089	1.837
Actions	Controls	1.222	0.107	152	1.026	1.455

Means, SEs, and confidence limits are back transformed to the original scale.

Among the covariates, age significantly predicted precision [*F*(1,55) = 5.35, *p* = 0.024], indicating that older participants showed lower WFs (higher precision). Mathematical ability also emerged as a significant predictor suggesting that higher math scores were associated with lower WFs [*F*(1,55) = 10.295, *p* = 0.002]. Verbal and non–verbal reasoning skills (Similarities and Matrices) did not show significant effects [*F*(1,55) = 0.376, *p* = 0.542, and *F*(1,55) = 1.65, *p* = 0.204, respectively]. Overall, these results suggest that although task demands and individual differences in age and mathematical ability had an influence on numerosity estimation precision, the diagnostic group status (ADHD vs. controls) did not.

### 4.2 Numerosity estimation accuracy

As before, we first evaluated a model predicting AB without including covariates (see Figure 2B for individual subject data). AB did not differ across tasks [*F*(2,122) = 1.56, *p* = 0.213], indicating that participants’ estimation error was similar across tasks. No group differences emerged. ADHD and control participants showed comparable levels of AB [*F*(1,61) = 0.968, *p* = 0.329]. On average, across tasks the AB in the ADHD group was 1.25, 95% CI = [1.08, 1.5], while in the control group was 1.15, 95%CI = [1.03, 1.29]. The corresponding LBF was −0.68 indicating substantial evidence for no group difference. The Task × Group interaction was also not significant [*F*(2,122) = 1.069, *p* = 0.347], indicating that this similarity held across all tasks, confirmed by post–hoc *t*–test (ADHD vs. Controls; Dots: *t*_(174)_ = −0.343, p_bonf_ = 1, d_adj =_ − 0.10, 95% CI = [−0.69, 0.48], LBF = − 0.88; Flashes: *t*_(174)_ = 0.77, p_bonf_ = 1, d_adj_ = 0.23, 95% CI = [−0.36, 0.81], LBF = −0.85; Actions: *t*_(174)_ = 1.53, p_bonf_ = 0.378, d_adj_ = 0.46, 95% CI = [−0.13, 1.04], LBF = −0.71).

The same model but including all the covariates confirmed the results (Table 2; Figure 3B). AB did not differ across tasks [*F*(2,118) = 1.75, *p* = 0.178], confirming that participants’ estimation error was similar across tasks. No group differences emerged. ADHD and control participants showed comparable levels of AB [*F*(1,55) = 0.044, *p* = 0.834], and the Task × Group interaction was not significant [*F*(2,118) = 0.95, *p* = 0.389], indicating that this similarity held across all tasks, confirmed by post–hoc *t*–test (ADHD vs. Controls; Dots: *t*_(137)_ = −0.69, p_bonf_ = 1, d_adj_ = −0.23, 95% CI = [−0.89, 0.43], LBF = −0.85; Flashes: *t*_(137)_ = 0.292, p_bonf_ = 1, d_adj_ = 0.10, 95% CI = [−0.56, 0.76], LBF = −0.89; Actions: *t*_(137)_ = 0.87, p_bonf_ = 1, d_adj_ = 0.29, 95% CI = [−0.37, 0.95], LBF = −0.82). Mathematical ability played a significant role. Higher math scores were associated with lower AB [*F*(1,55) = 4.98, *p* = 0.03], indicating more accurate estimations. In contrast, age and the reasoning skills (Similarities and Matrices) were not significant predictors [*F*(1,55) = 0.01, *p* = 0.9, *F*(1,55) = 0.002, *p* = 0.96, and *F*(1,55) = 0.368, *p* = 0.54, respectively]. Taken together, these findings indicate that accuracy (like precision) is primarily influenced by individual differences in mathematical ability rather than by diagnostic group status.

### 4.3 Temporal characteristics of the motor sequences

The results revealed group differences in motor timing, that were consequently inherited by the motor–derived visual sequences (see methods). Participants with ADHD showed a higher mean tapping rate than controls (ADHD: M = 4.6 Hz, SD = 1.27; controls: M = 3.72 Hz, SD = 0.9 Hz, *t*_(61)_ = 3.12, *p* = 0.003, Cohen d = 0.84). Mean sequence duration was shorter in the ADHD group (M = 3.04 s, SD = 0.75 s) than in the control group (M = 3.7 s, SD = 0.84 s, *t*_(61)_ = −2.97, *p* = 0.004, Cohen d = −0.8). In contrast, the groups did not differ in IEI coefficient of variation (ADHD: M = 0.436, SD = 0.294; controls: M = 0.409, SD = 0.320, *t*_(61)_ = 0.31, *p* = 0.756, Cohen d = 0.08). Overall, participants with ADHD produced faster and shorter motor sequences, and these characteristics resulted in correspondingly faster and shorter visual flash sequences. However, the relative temporal regularity of both the motor and derived visual sequences was comparable between groups.

### 4.4 Analysis excluding dyscalculia

To assess whether the main findings were influenced by comorbid dyscalculia, we repeated the covariate–adjusted analyses after excluding the three ADHD participants with dyscalculia. The overall pattern remained unchanged. For estimation precision, neither the effect of Group, *F*(1,52) = 0.126, *p* = 0.724, nor the interaction between Task and Group, *F*(2,112) = 0.121, *p* = 0.886, was significant. Similarly, for accuracy, neither the effect of Group, *F*(1,52) = 0.006, *p* = 0.94, nor the Task by Group interaction, *F*(2,112) = 1.32, *p* = 0.269, was significant. Task–specific ADHD–control contrasts were also non–significant for both outcomes (minimum uncorrected *p* value = 0.126). These results suggest that the overall pattern of findings was not driven by the three participants with dyscalculia.

## 5 Discussion

The main aim of the current study was to investigate numerosity verbal estimation in children with ADHD and controls across space and time (dot arrays, temporal sequences) and sensory modalities (visual flashes, hand actions). Despite lower mathematical performance compared to controls, we found no clear evidence of numerosity estimation impairment in this sample with ADHD. More specifically, the findings for estimation precision were model–dependent. The unadjusted analysis indicated lower overall precision in the ADHD group (mean Wf = 0.188, compared with 0.16 in controls). However, the Bayesian evidence for this overall group difference was inconclusive. Moreover, none of the task–specific contrasts survived Bonferroni correction, and the task-specific Bayes factors favoured the null hypothesis, although the evidence was anecdotal for dots and flashes and substantial for actions. The group difference disappeared after adjustment for age, reasoning abilities, and mathematical performance. No overall or task–specific group differences emerged for estimation accuracy (absolute bias) in either the unadjusted or adjusted analyses.

These findings confirm and expand previous work (Anobile et al., 2022). In that earlier investigation, using a comparison task the authors found evidence for unimpaired visual numerosity processing in ADHD. That paradigm required participants to compare visual arrays of dots without any mapping between numerosity and symbolic numbers. In the current study, participants were instead asked to verbally estimate numerosity, thus requiring an explicit mapping between quantity representations and their symbolic verbal counterpart. Despite this additional representational demand, children with ADHD showed no clear evidence of impaired performance, suggesting a preserved visual spatial number sense. The current study extends this conclusion well beyond the visual–spatial domain. No clear evidence for a deficit was also observed for temporal visual sequences (flashes) and self–generated motor actions (button presses). To our knowledge, this broader cross–domain extension is novel and indicates that the previously observed absence of an ADHD–control difference is not restricted to visual–spatial comparison, extending across different tasks and numerical formats, spanning space, time, vision, and action. Many studies observed visual spatial numerosity deficits in dyscalculia, linking this neurodevelopmental disorder to an early impairment in the visual number sense (Anobile et al., 2018b; Decarli et al., 2023; Mazzocco et al., 2011; Piazza et al., 2010). Within that framework, impaired representation of approximate numerical magnitude is thought to constrain the later development of formal mathematics (Piazza, 2010). The current findings do not support a comparable account for ADHD. Although children with ADHD showed lower mathematical performance than controls, this was not mirrored by impaired numerosity processing. Taken together with previous findings, the current results suggest that the mathematical difficulties associated with ADHD are unlikely to arise from a primary deficit in the basic number sense. One possibility is that the mathematical difficulties observed in ADHD are linked to domain–general factors, such as attentional control, working memory, executive functioning or response monitoring rather than to a domain–specific impairment in representing quantity. This possibility fits well with a large body of literature suggesting a key role of executive functions in mathematical learning (Friso-van den Bos et al., 2013; Peng et al., 2016; Purpura et al., 2017; Ribner et al., 2026; Spiegel et al., 2021; Zhu et al., 2024). A similar pattern of results has been reported in individuals born preterm. While prematurity is a well–known risk factor for mathematical learning disorders (De Rodrigues et al., 2006; Taylor et al., 2009), both school age children (Tinelli et al., 2015) and newborns (Anobile et al., 2021b) born preterm do not show numerosity deficits, again suggesting a different aetiology compared to dyscalculia.

The current study has limitations. The relatively small ADHD sample may have limited sensitivity to small or moderate group differences. The Bayes factors were generally consistent with the absence of group differences, although the strength of the evidence varied across analyses. Accordingly, the present findings should be interpreted as showing no clear evidence of impaired numerosity estimation precision or accuracy in this sample, rather than as demonstrating a fully preserved numerosity processing in ADHD, more generally. Larger samples are needed to generalize the findings and to exclude small–to–moderate impairments. It is nevertheless noteworthy that the same sample showed a clear group difference in mathematical ability and reasoning measures, indicating that between–group differences could be detected on some outcomes. However, sensitivity may vary across measures because of differences in variability and measurement precision. Given the limited sample size, we adopted a parsimonious model that did not include higher–order interactions. Consequently, whether the association between mathematical skills and numerosity performance differs between groups or across psychophysical tasks remains an open question. The limited sample size also precluded adequately powered analyses stratified by ADHD presentation or sex, as the resulting subgroups would have included too few participants. These factors may therefore limit the generalizability of the findings across ADHD presentations and across sexes. We also did not examine the influence of attentional and executive functions. Future studies should investigate more directly whether the mechanisms linking ADHD to poorer mathematical achievement primarily reflect attentional and/or executive factors, and how these pathways may differ from those involved in dyscalculia, with or without comorbid ADHD. A further potential limitation concerns the individualized temporal structure of the visual stimuli. Because each participant–specific flash sequence reproduced the temporal characteristics of the corresponding motor sequence, the visual sequences were not matched for rate and duration across groups. Participants with ADHD produced faster and shorter motor sequences than controls, and these differences were therefore retained in the visual stimuli. However, the groups did not differ in IEI coefficient of variation, indicating comparable relative temporal regularity. Although estimation precision and accuracy in the flash task were comparable between groups, the current design cannot isolate the potential contribution of stimulus rate and duration to visual–temporal numerosity estimation. Finally, despite the provision of breaks, the relatively long testing session (approximately 1.5 h) may have introduced fatigue–related effects that should be considered when interpreting the current findings.

In conclusion, the current study extends previous evidence by showing no clear evidence of impaired numerosity processing in children with ADHD across different tasks (discrimination vs. estimation) and stimuli formats (visual–spatial, visual–temporal, and motor–temporal). Thus, unlike what has often been observed in dyscalculia, the present findings do not support the hypothesis that mathematical difficulties associated with ADHD primarily arise from an impairment in the basic number sense. However, larger studies are needed before concluding that numerosity processing is fully preserved in ADHD.

## Data Availability

The raw data supporting the conclusions of this article will be made available by the authors, without undue reservation.
